# Radiative Transfer and Generalized Wind

**DOI:** 10.3390/e22101153

**Published:** 2020-10-14

**Authors:** Christopher Essex, Indrani Das

**Affiliations:** Department of Applied Mathematics, the University of Western Ontario, London, ON N6A 5B7, Canada; idas2@uwo.ca

**Keywords:** entropy production, radiative energy transfer, radiative entropy transfer, two-stream grey atmosphere, energy flux density, entropy flux density, generalized winds

## Abstract

Dissimilar flows can be compared by exploiting the fact that all flux densities divided by their conjugate volume densities form velocity fields, which have been described as generalized winds. These winds are an extension of the classical notion of wind in fluids which puts these distinct processes on a common footing, leading to thermodynamical implications. This paper extends this notion from fluids to radiative transfer in the context of a classical two-stream atmosphere, leading to such velocities for radiative energy and entropy. These are shown in this paper to exhibit properties for radiation previously only thought of in terms of fluids, such as the matching of velocity fields where entropy production stops.

## 1. Introduction

We understand wind as a phenomenon in a gas or plasma where an impulsive load is delivered by the anisotropic distribution of individual particle velocities realized in the form of a vector field stemming from the collective behaviour of gas particles. Currents in liquids might also be termed “winds” in a general sense. The mechanical wind velocity field can similarly be thought of as the mass flux, ρv, divided by the volume density of mass ρ. A mechanical wind vector field is the central product of fluid mechanics, typically extracted via the Navier–Stokes equations. Its solutions form a vector field of velocities with a classical rest frame.

The wind velocity can also be seen as implying a rest frame without wind. In that rest frame, the fluid is at rest and local thermodynamic conditions can then be considered. However, out of full thermodynamic equilibrium, that frame does not truly represent a state of rest. There are still currents of other thermodynamical and dynamical physical properties passing though that frame, like energy and momentum to mention a few. These velocity fields also represent frames. An observer riding with the classical wind is not at rest in these other frames.

These fluxes each have different units and as such are difficult to compare, unless one observes that each flux density, G, and its associated volume density, ϕ, induces a velocity vector field of its own, $v_{\phi }=G/\phi$. S. Sieniutycz [1,2] observed that all such vector fields must become identical in thermodynamic equilibrium. He used this insight to explore covariant fluid dynamics and thermodynamics. Each of these fluxes inducing its own vector field, implies a distinct rest frame. When all of these vector fields are the same, there is one reference an observer could ride in where all currents stop. The term “generalized wind” was later coined to describe these vector fields [3]. When a single “generalized wind” velocity exists for all currents there exists a frame where no process occurs. The one-frame condition for all winds becomes a necessary condition for thermodynamic equilibrium. This implies that the entropy production rate must vanish when all generalized wind frames agree.

Fluid dynamics are normally considered distinct from radiative transfer. However, they both can be traced to a common structure in momentum space, wherein the total time derivative of the mean occupation number n(r(t),p(t),t) leads to the primary transport equations for both fluids and radiative transfer. The outcomes through their respective moment equations are very different however. The classical Navier–Stokes equations can be seen as providing rest frames, while radiative transfer concerns photons with no rest frame. Generalized winds must exist for photons too in radiative transfer. There is an energy velocity, $v_{e}=F/u$ stemming from the energy flux density, F, and the volume radiation energy density, *u*. There is also an entropy velocity, $v_{s}=H/s_{r}$, arising from the entropy flux density, **H**, and the entropy volume density, $s_{r}$. This paper addresses whether these generalized winds for radiation follow the prescription that velocity fields must agree when entropy production is zero and not elsewhere.

The static plane parallel grey atmosphere was instrumental in the development of the theory of integral equations in the first half of the twentieth century by Hopf, Milne, Schwarzschild and others and of course essential to astrophysics. In this regard, this is a classical subject, but the notion of generalized winds has not been considered. This paper explores the extent to which $v_{e}$ and $v_{s}$ follow the necessary conditions on the entropy production rate in a classical plane parallel, two-stream atmosphere, while considering the notion of rest frames for generalized winds. This paper shows that the thermodynamical expectations for radiation generalized winds hold within the limitations of the simplified classical radiative transfer problem.

## 2. Preliminaries

This section has two goals. It lays down the radiative transfer framework for those unfamiliar with the subject, and it introduces radiative entropy transfer for those already familiar with radiative transfer of energy. We proceed in a parallel manner between energy and entropy in order to highlight the close parallels intuitively. This representation is used where possible throughout our paper.

### 2.1. Energy and Entropy Radiation: The Specific Intensities, Fluxes and Volume Densities

We proceed from the mean occupation number, but expressed in terms of a time-varying number flux density per unit volume and unit solid angle defined in terms of position, frequency and direction: $n(r\left(t\right),\nu ,\hat{m},t)$ [4]. It is straightforward to connect this primary statistical mechanical object to the specific intensity (also called radiance) which is energy flux density per unit solid angle and frequency, and similarly for entropy. Thus, the frequency dependent specific intensity for photon energy, $I_{\nu }$, and the same for entropy, $J_{\nu }$, are,

(1) $$\begin{array}{c} I_{\nu }=\frac{2h\nu^{3}}{c^{2}}n\;;\;J_{\nu }\equiv \frac{2k_{B}\nu^{2}}{c^{2}}\left[\left(1+n\right)\ln\left(1+n\right)-n\ln n\right]. \end{array}$$

The factor of 2 in each indicates unpolarised radiation. The entropy expression comes directly from counting Bosons [4].

Moment integrals, in $\hat{m}$, over all solid angles and frequencies provide well-known integrals for volume densities and flux densities for both energy and entropy, respectively,

(2) $$\begin{array}{c} u=\frac{1}{c}\int I_{\nu }\;d\Omega d\nu \;;\;s_{r}=\frac{1}{c}\int J_{\nu }\;d\Omega d\nu \end{array}$$

where *c* is the speed of light.

Accordingly, vector energy flux density (F) and entropy flux density (H) represent first moments,

(3) $$\begin{array}{c} F=\int I_{\nu }\hat{m}\;d\Omega d\nu \;;\;H=\int J_{\nu }\hat{m}\;d\Omega d\nu \end{array}$$

where F and H are presented as the first order moment of the corresponding energy and entropy density function. Note that (2) represents the zeroth order moment of their respective density function.

### 2.2. Radiative Energy and Entropy Transfer Equations

The equation of radiative energy and entropy transfer can simply be written in the following differential form:

(4) $$\begin{array}{c} \frac{1}{c}\frac{\partial I_{\nu }}{\partial t}+\hat{m}\cdot \nabla I_{\nu }\;=-\kappa_{\nu }I_{\nu }+j_{\nu }\;;\;\frac{1}{c}\frac{\partial J_{\nu }}{\partial t}+\hat{m}\cdot \nabla J_{\nu }\;=-\kappa_{\nu }J_{\nu }+i_{\nu } \end{array}$$

These are easily deduced by differentiating the mean occupation number in time and then applying (1). The right sides are general if $j_{\nu }$ and $i_{\nu }$ are left unspecified. Kirchhoff’s law is easily generalized to include entropy too by considering entropy transfer in (4) and considering the equilibrium case when derivatives vanish leaving,

(5) $$\begin{array}{c} j_{\nu }=\kappa_{\nu }B_{\nu }\;;\;i_{\nu }=\kappa_{\nu }L_{\nu } \end{array}$$

where in thermodynamic equilibrium, $I_{\nu }=B_{\nu }$, the Planck function intensity, and $J_{\nu }=L_{\nu }$, the intensity of the equilibrium entropy distribution corresponding to the Planck function. That latter can be easily determined through (1).

Classical radiative transfer due to Kirchhoff [5] holds that the equilibrium value for $i_{\nu }$ holds out of equilibrium too. One may make a similar claim for entropy, $j_{\nu }$. Both assumptions fail in the small ν limit on account of stimulated emission, but hold very well generally simultaneously [6].

Now, integrating (4) over the entire range of frequencies and solid angles and using (2) and (3), one obtains

(6) $$\begin{array}{c} \frac{\partial u}{\partial t}+\nabla \cdot F=\epsilon \;;\;\frac{\partial s_{r}}{\partial t}+\nabla \cdot H=\xi \end{array}$$

where ϵ and ξ present the source strengths for energy and entropy radiation, respectively.

In the latter case, if radiative absorption and emission are the only irreversible processes, as it is in classical radiative transfer, then ξ is the entropy production rate and ξ>0 according to the second law of thermodynamics. Energy radiation under similar conditions is in radiative equilibrium, i.e., ϵ=0 or ∇·F=0 under steady conditions.

### 2.3. Classical Grey RT in Plane Parallel Geometry under Steady State Conditions

For simplicity, we proceed with a classical steady state grey atmosphere [5,7]. In keeping with the classical picture, we employ a plane parallel geometry. Then, (4) and (5) are reduced to the following form,

(7) $$\begin{array}{c} \mu \frac{dI_{\nu }}{dz}\;=-\kappa_{\nu }I_{\nu }+\kappa_{\nu }B_{\nu }\;;\;\mu \frac{dJ_{\nu }}{dz}\;=-\kappa_{\nu }J_{\nu }+\kappa_{\nu }L_{\nu } \end{array}$$

where μ is the direction cosine from the spherical geometry of the moment integrals, arising from $\hat{m}\cdot \hat{k}$, where $\hat{k}$ is the upward direction vector in the atmosphere. Recall that the plane parallel geometry has only one meaningful dimension. On symmetry grounds we find that $F=F\;\hat{k}$, and $H=H\;\hat{k}$. Thus,

(8) $$\begin{array}{c} F=\int I_{\nu }\;\mu \;d\Omega \;d\nu \;;\;H=\int J_{\nu }\;\mu \;d\Omega \;d\nu \end{array}$$

The grey approximation means that the volume absorption coefficient, $\kappa_{\nu }$, has no dependence on frequency ν. Thus, $\kappa_{\nu }\Rightarrow \kappa .$ We introduce the optical depth in the classical way to employ a coordinate system natural to the physical process in radiative transfer. τ=0 is the top of the atmosphere and it increases with decreasing altitude, *z*.

(9) $$\begin{array}{c} d\tau =-\kappa dz \end{array}$$

Returning to (7), with these conditions and definitions, we find the classical result for energy and a close analogue for entropy,

(10) $$\begin{array}{c} \mu \frac{dI}{d\tau }\;=I-B\;;\;\mu \frac{dJ}{d\tau }\;=J-L. \end{array}$$

Here, *I* and *J* are the frequency integrated specific intensity for energy and entropy, respectively. *B* and *L* can be found by integrating $B_{\nu }$ and $L_{\nu }$ over all frequencies. These too are well known [4],

(11) $$\begin{array}{c} B=\int_{0}^{\infty }B_{\nu }d\nu =\frac{\sigma }{\pi }T^{4}\;;\;L=\int_{0}^{\infty }L_{\nu }d\nu =\frac{4}{3}\frac{\sigma }{\pi }T^{3} \end{array}$$

where σ is the Stefan–Boltzmann constant. One can relate *B* and *L* in the following way,

(12) $$\begin{array}{c} \frac{4}{3}{\left(\frac{\sigma }{\pi }\right)}^{\frac{1}{4}}B^{\frac{3}{4}}=L \end{array}$$

### 2.4. Moment Equations and Radiative Equilibrium

Integrating (10) over all solid angles, one obtains

(13) $$\begin{array}{c} \frac{dF}{d\tau }=cu-4\pi B\;;\;\frac{dH}{d\tau }=cs_{r}-4\pi L \end{array}$$

We write the radiation energy pressure per volume, *P*, and entropy pressure analogue, *R*, in as the second moment of respective intensities,

(14) $$\begin{array}{c} P=\frac{1}{c}\int I\mu^{2}d\Omega \;;\;R=\frac{1}{c}\int J\mu^{2}d\Omega \end{array}$$

Multiplying (10) by μ, integrating over solid angle, then using the definitions (14) yields,

(15) $$\begin{array}{c} \frac{dP}{d\tau }=\frac{1}{c}F\;;\;\frac{dR}{d\tau }=\frac{1}{c}H \end{array}$$

Radiative equilibrium requires, $\epsilon =0\;\Rightarrow \;\frac{dF}{d\tau }=0$. It breaks the parallelism between energy and entropy radiation as $\frac{dH}{d\tau }=\xi >0$ by the second law of thermodynamics. The energy equations significantly simplify in radiative equilibrium. That is exemplified by (13), which becomes

(16) $$\begin{array}{c} u=\frac{4\pi B}{c} \end{array}$$

### 2.5. Classical Two Stream Atmosphere with Entropy Radiation

Further progress is made with the classical two-stream assumptions. When moments are taken, instead of assuming that the intensities vary with μ we assume that they are independent of μ, except that intensities only differ between the upper and lower hemispheres (e.g., [7]). The problem becomes a complex computational problem without this assumption, which will be explored in future work. We may use this approximation here to eliminate *P* and *R* from (15). First we find

(17) $$\begin{array}{c} u=\frac{2\pi }{c}\left(I^{+}+I^{-}\right)\;;\;s_{r}=\frac{2\pi }{c}\left(J^{+}+J^{-}\right) \end{array}$$

(18) $$\begin{array}{c} F=\pi \left(I^{+}-I^{-}\right)\;;\;H=\pi \left(J^{+}-J^{-}\right) \end{array}$$

(19) $$\begin{array}{c} P=\frac{2\pi }{3c}\left(I^{+}+I^{-}\right)\;;\;R=\frac{2\pi }{3c}\left(J^{+}+J^{-}\right) \end{array}$$

where the upward intensities of *I* and *J* are denoted by $I^{+}$ and $J^{+}$, respectively, while the downward ones are denoted by $I^{-}$ and $J^{-}$, respectively.

An interesting outcome emerges in passing, as we know radiation pressure volume density is one-third of energy volume density in thermodynamic equilibrium. This emerges for our two-stream atmosphere too, as well as, intriguingly, for entropy,

(20) $$\begin{array}{c} P=\frac{u}{3}\;;\;R=\frac{s_{r}}{3}. \end{array}$$

Differentiating (20) with respect to τ then using (15) yields

(21) $$\begin{array}{c} \frac{du}{d\tau }=\frac{3}{c}F\;;\;\frac{ds_{r}}{d\tau }=\frac{3}{c}H \end{array}$$

Differentiating (13) w.r.t. τ and using (21) gives,

(22) $$\begin{array}{c} \frac{d^{2}F}{d\tau^{2}}-3F=-4\pi \frac{dB}{d\tau }\;;\;\frac{d^{2}H}{d\tau^{2}}-3H=-4\pi \frac{dL}{d\tau } \end{array}$$

Differentiating the latter part of (21) w.r.t. τ, and substituting the corresponding part from (13) yields,

(23) $$\begin{array}{c} \frac{d^{2}s_{r}}{d\tau^{2}}-3s_{r}=-\frac{12\pi }{c}L \end{array}$$

In radiative equilibrium (22) yields,

(24) $$\begin{array}{c} \frac{dB}{d\tau }=\frac{3}{4\pi }F\Rightarrow B=\frac{3}{4\pi }F\tau +B\left(0\right) \end{array}$$

At τ=0, $I^{-}=0$; thus, (16) through (18) require $\frac{F}{2\pi }=B\left(0\right)$ or

(25) $$\begin{array}{c} B=\frac{F}{2\pi }\left(\frac{3}{2}\tau +1\right) \end{array}$$

and substituting into (12) gives,

(26) $$\begin{array}{c} L=\frac{4}{3}{\left(\frac{\sigma }{\pi }\right)}^{1/4}{\left(\frac{F}{2\pi }\right)}^{3/4}{\left[\frac{3}{2}\tau +1\right]}^{3/4} \end{array}$$

As τ increases, the radiation state approaches thermodynamic equilibrium. The field approaches isotropy and the energy intensity approaches the ambient black body function, *B*. We see this by using (25) and by breaking up (16) through (18) to find $I^{+}$ and $I^{-}$ as following

(27) $$\begin{array}{c} I^{+}=B\left(\frac{\frac{3}{2}\tau +2}{\frac{3}{2}\tau +1}\right)\;;\;I^{-}=B\left(\frac{\frac{3}{2}\tau }{\frac{3}{2}\tau +1}\right) \end{array}$$

As τ increases both intensities become the same and approach the integrated Planck function.

This is a key observation because it implies that we must require that $J^{+}\to J^{-}\to L$ in the large τ limit. Similarly H→0 and $cs_{r}\to 4\pi L$ and their derivatives above (see (13) and (21)) vanish in that limit.

## 3. Generalized Winds

A vector velocity field is associated with a vector flux density and a scalar volume density. This vector field is defined as a generalized wind, which is simply the ratio of a flux density with its corresponding volume density. Thus, radiative energy velocity ($v_{e}$) and entropy velocity ($v_{s}$) are given by,

(28) $$\begin{array}{c} v_{e}=\frac{F}{u}\;;\;v_{s}=\frac{H}{s_{r}} \end{array}$$

Simplifying using (27), we find

(29) $$\begin{array}{c} v_{e}=\frac{F}{u}=\frac{c}{3\tau +2} \end{array}$$

Solving for $v_{s}$ is more complicated. From above (see the latter part of (13) and (21) each) we have the coupled system as follows,

(30) $$\begin{array}{c} \left(\begin{array}{c} H^{\prime }\\ cs_{r}^{\prime } \end{array}\right)=\left(\begin{array}{cc} 0 & 1\\ 3 & 0 \end{array}\right)\left(\begin{array}{c} H\\ cs_{r} \end{array}\right)-4\pi L\left(\begin{array}{c} 1\\ 0 \end{array}\right) \end{array}$$

where $H^{\prime }$ and $s_{r}^{\prime }$ denote derivatives of *H* and $s_{r}$ w.r.t. τ, respectively.

After diagonalization of the above, (30) gives

(31) $$\begin{array}{c} \left(\begin{array}{c} z_{1}^{\prime }\\ z_{2}^{\prime } \end{array}\right)=\left(\begin{array}{cc} \sqrt{3} & 0\\ 0 & -\sqrt{3} \end{array}\right)\left(\begin{array}{c} z_{1}\\ z_{2} \end{array}\right)-4\pi L\left(\begin{array}{c} 1\\ 1 \end{array}\right) \end{array}$$

where $z_{1}=\left(H+\frac{cs_{r}}{\sqrt{3}}\right)$ and $z_{2}=\left(H-\frac{cs_{r}}{\sqrt{3}}\right)$. $z_{1}^{\prime }$ and $z_{2}^{\prime }$ denote derivatives of $z_{1}$ and $z_{2}$ w.r.t. τ, respectively.

Now, solving (31) for $z_{1}$ and $z_{2}$ yields,

(32) $$\begin{array}{c} \left(\begin{array}{c} z_{1}\\ z_{2} \end{array}\right)=\left(\begin{array}{c} -4\pi \int_{0}^{\tau }Le^{-\sqrt{3}\left(t-\tau \right)}dt+z_{1}\left(0\right)e^{\sqrt{3}\tau }\\ -4\pi \int_{0}^{\tau }Le^{\sqrt{3}\left(t-\tau \right)}dt+z_{2}\left(0\right)e^{-\sqrt{3}\tau } \end{array}\right) \end{array}$$

In the above equation, there are two conditions that define $z_{1}\left(0\right)$ and $z_{2}\left(0\right)$. The first condition is that at the top of the atmosphere $J^{-}=0$. Using the two stream definitions above (see Section 2.5) this implies that $H\left(0\right)=cs_{r}\left(0\right)/2$. Thus, it follows

(33) $$\begin{array}{c} \left(\begin{array}{c} z_{1}\\ z_{2} \end{array}\right)=\left(\begin{array}{c} -4\pi \int_{0}^{\tau }Le^{-\sqrt{3}\left(t-\tau \right)}dt+H\left(0\right)\left(1+\frac{2}{\sqrt{3}}\right)e^{\sqrt{3}\tau }\\ -4\pi \int_{0}^{\tau }Le^{\sqrt{3}\left(t-\tau \right)}dt+H\left(0\right)\left(1-\frac{2}{\sqrt{3}}\right)e^{-\sqrt{3}\tau } \end{array}\right) \end{array}$$

The other physical condition on the atmosphere is that thermodynamic equilibrium must be approached asymptotically with optical depth. This is realized by an asymptotic approach to zero of $\frac{dH}{d\tau }$ and $\frac{ds_{r}}{d\tau }$ with increasing τ. That implies, using $z_{1}$, that $z_{1}^{\prime }\to 0,$ as τ grows. Thus,

(34) $$\begin{array}{c} H\left(0\right)∼\frac{4\pi }{1+\frac{2}{\sqrt{3}}}\int_{0}^{\tau }Le^{-\sqrt{3}t}dt\Rightarrow H\left(0\right)=\frac{4\pi }{1+\frac{2}{\sqrt{3}}}\int_{0}^{\infty }Le^{-\sqrt{3}t}dt \end{array}$$

This means that the entropy flux at the top of the atmosphere is the sum of the entropy emissions from the whole atmosphere.

**Figure 1:**
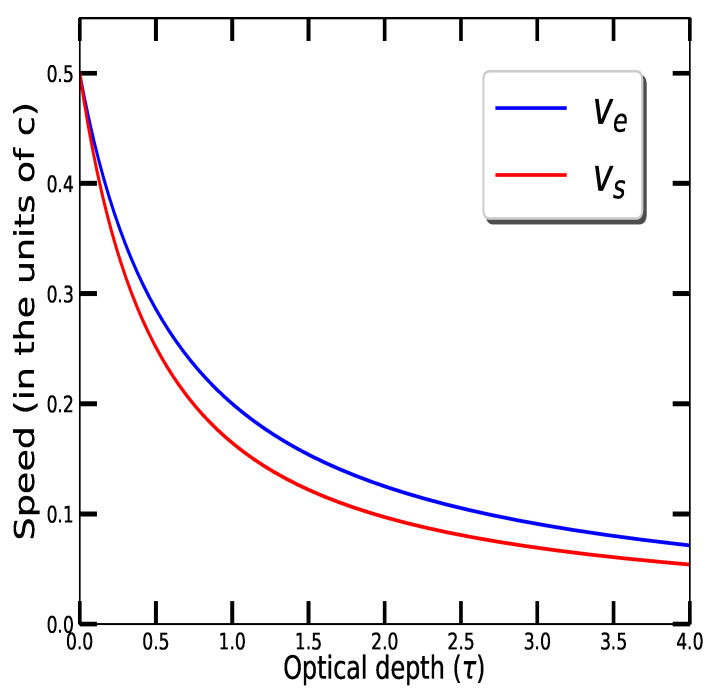
Energy speed ($v_{e}$) and entropy speed ($v_{s}$) as a function of optical depth (τ). Both of these are evaluated in the units of speed of light (*c*).

Finally, we obtain

(35) $$\begin{array}{c} v_{s}=\frac{c}{\sqrt{3}}\frac{z_{1}+z_{2}}{z_{1}-z_{2}}=\frac{c}{\sqrt{3}}\frac{\left\{-\chi \left(\tau \right)+\chi \left(\infty \right)\right\}e^{\sqrt{3}\tau }+\left\{-\psi \left(\tau \right)+\chi \left(\infty \right)\frac{\sqrt{3}-2}{\sqrt{3}+2}\right\}e^{-\sqrt{3}\tau }}{\left\{-\chi \left(\tau \right)+\chi \left(\infty \right)\right\}e^{\sqrt{3}\tau }-\left\{-\psi \left(\tau \right)+\chi \left(\infty \right)\frac{\sqrt{3}-2}{\sqrt{3}+2}\right\}e^{-\sqrt{3}\tau }} \end{array}$$

where $\chi \left(\tau \right)=\int_{0}^{\tau }{\left[\frac{3}{2}t+1\right]}^{3/4}e^{-\sqrt{3}t}dt$ and $\psi \left(\tau \right)=\int_{0}^{\tau }{\left[\frac{3}{2}t+1\right]}^{3/4}e^{\sqrt{3}t}dt$ arriving at the curious fact that both $v_{s}$ and $v_{e}$ are functions of τ only. Thus, Figure 1 is unchanged no matter what atmosphere it represents: planetary atmosphere or star!

The speeds of the generalized winds are plotted in Figure 1 using (29) and (35). The top of the atmosphere (i.e., up) is at the left (τ=0) and the atmosphere’s interior (i.e., down) is off to the right—truncated at τ=4 in Figure 1 for convenience. This configuration of coordinates is the standard in radiative transfer.

In Figure 1 we see that both $v_{e}$ and $v_{s}$ increase with altitude. This might be interpreted as the result of a net force if we were considering bodies with mass. No such mechanical thinking is appropriate here. The very general picture of equilibrium presented in the introduction does not require such mechanics to be in play.

Off of the right side of the figure, the velocities are both asymptotically approaching zero as densities increase while *F* is a constant and *H* is decreasing to zero. Thermodynamic conditions approach equilibrium in that limit too, agreeing with expectations that $v_{e}\to v_{s}$ in that limit. Thus, we expect that the blue and red curves will grow apart with decreasing optical depth. This will continue until the influences of the top of the atmosphere are encountered. Classically this is expected to occur at optical depth 1 or so. Indeed, this appears to be so in Figure 1 where $v_{e}-v_{s}$ begins to decrease with decreasing optical depth near τ=1. The top of the atmosphere τ=0 is where irreversible absorption and re-emission stops in the absence of any absorbing material, and so we expect that $v_{e}=v_{s}$.

It is remarkable that this result is independent of the particulars of any atmosphere. That is, these functions hold for a two-stream approximation for any star or planet. The special case of a finite optical depth only requires an energy and entropy flux to be supplied at the largest τ.

## 4. Conclusions

This paper provided a quick introduction to classical radiative transfer while appending a new parallel development for radiative entropy transfer. This allowed us to address the classical plane parallel, two-stream, grey atmosphere in a new way. That geometry is applicable to both stellar and planetary atmospheres. With this foundation we were able to turn to the thermodynamics question; in particular, the proposition that all generalized wind vector fields must become identical in thermodynamic equilibrium, or at least in the absence of irreversible processes.

The results within the two-stream assumption strongly suggest that this concept holds for radiation too, extending it beyond its origins in fluid mechanics. The entropy production in this paper does indeed stop at the top of the atmosphere where its generalized winds become the same. Similarly, in the infinite optical depth limit, where equilibrium is approached, the velocities become the same too.

There are however a number of questions. Some need to be addressed in a full, non-two-stream treatment. Some are simpler than others. One of these is the significance of the speed c/2 at the top of the atmosphere. Is this top speed the same value in the full non-two-stream treatment? Another question concerns where the largest separation in the functions $v_{s}$ and $v_{e}$ occurs at τ≈1. Is there a maximum in the entropy production rate there? The functions $v_{s}$ and $v_{e}$ are invariant across stellar and planetary atmospheres. Is this true for a full non-two-stream treatment too?

A convenience in thinking about congruent vector fields is the existence of a rest frame that one can imagine travelling with the flow on. While this is easily imagined with vector fields of nearly zero magnitude in the deep interior of the atmosphere, there clearly is a problem at the top of the atmosphere. There is no rest frame there, because there is no radiation coming down to be blue shifted there and the rest of the radiation is red shifted, even that coming from the sides because of time dilation. Photons have no rest frame, but radiation flows can have one. Is there a transition between a rest-frame flow and a more streaming-like flow without one? At what τ would the transition occur? Is it connected with optical depth 1? Is the existence of a rest frame necessary to the concept?

Additionally, we have not even considered the role of scattering which has rich complications as well. These are all interesting questions that our forthcoming work will address.
